# Knowledge, attitudes, and practices (KAP) toward COVID-19: a cross-sectional study in South Korea

**DOI:** 10.1186/s12889-021-10285-y

**Published:** 2021-02-05

**Authors:** Minjung Lee, Bee-Ah Kang, Myoungsoon You

**Affiliations:** 1 Department of Public Health Sciences, Graduate School of Public Health, Seoul National University, 1 Gwanak-ro, Gwanak-gu, Seoul, 08826 South Korea; 2 Office of Dental Education, School of Dentistry, Seoul National University, Seoul, South Korea; 3 Department of Communication, Seoul National University, 1 Gwanak-ro, Gwanak-gu, Seoul, 08826 South Korea; 4 Institute of Health and Environment, Seoul National University, 1 Gwanak-ro, Gwanak-gu, Seoul, 08826 South Korea

**Keywords:** COVID-19, Knowledge, Attitude, Practice, Survey, South Korea, Public health

## Abstract

**Background:**

The public must routinely practice precautionary behaviors to control the spread of COVID-19, as no vaccines and antiviral treatments are currently available. This paper examines the public’s knowledge, attitudes, and practices (KAP) related to COVID-19 and their relationships and identified the pandemic’s vulnerable populations to provide recommendations for behavioral interventions and policies.

**Methods:**

Data collection took place over 3 days (June 26–29) via an online survey 5 months after the Korea Centers for Disease Control and Prevention (KCDC) confirmed the first COVID case in South Korea; 970 subjects were included in the statistical data analysis.

**Results:**

Knowledge directly affected both attitudes (e.g., perceived risk and efficacy belief) and practices (e.g., personal hygiene practices and social distancing). Among the influencing factors of COVID-19 preventive behaviors, efficacy belief was the most influential and significant practice factor. It mediated the relationship between knowledge and all three preventive behaviors (wearing facial masks, practicing hand hygiene, and avoiding crowded places). The level of knowledge varied by sociodemographic characteristics. Females (*β* = 0.06, *p* <  0.05) and individuals with higher levels of education (*β* = 0.06, *p* <  0.05) demonstrated higher levels of knowledge.

**Conclusion:**

To increase precautionary behaviors among the public, health officials and policymakers must promote knowledge and efficacy belief. Future interventions and policies should also be developed in a ‘person-centered’ approach, targeting vulnerable subgroups, embracing them, and closing the gap of KAP toward COVID-19.

**Supplementary Information:**

The online version contains supplementary material available at 10.1186/s12889-021-10285-y.

## Background

Since many cases of the novel coronavirus disease 2019 (COVID-19) first appeared in Wuhan, China, in December 2019 [1, 2], the virus has infected millions worldwide. On January 30, 2020, the World Health Organization (WHO) declared that the outbreak of COVD-19 constituted a Public Health Emergency of International Concern (PHEIC), calling for countries to take urgent and aggressive action against the spread of the virus [3]. The epidemic in South Korea is an unprecedented crisis in recent history. After the first case was announced on January 20, there have been 59,773 confirmed cases and 879 deaths as of December 30, 2020 [4]. Given the epidemic’s scale, timing, and unpredictability threatening the health care system’s routine capabilities, South Korea is confronting a public health emergency [5, 6] and experiencing an ongoing battle with the virus.

Responding to the pandemic has become a serious challenge, as little is known about the epidemiological evidence of the disease, including its transmission dynamics, epidemic doubling time, and reproductive frequency [1]. Also, there are no vaccines or treatments clinically proven to be effective yet. With the scarcity of clinical measures raising heightened concerns, it becomes increasingly essential for the public to engage in precautionary behaviors and the disease response and surveillance efforts at the policy level [5–7]. Amidst pandemics, educating, engaging, and mobilizing the public to become active participants may help achieve public health emergency preparedness, reducing the overall population’s vulnerability [6]. When people collectively engage in preventive behaviors, including practicing personal hygiene and maintaining social distance, it is possible to control the spread of the disease, according to recent studies highlighting that individual behaviors may dramatically decrease morbidity and mortality rates of COVID-19 [7, 8]. Therefore, a routine practice of precautionary behaviors among the public must become the new status quo.

For non-pharmaceutical public health interventions to successfully encourage and sustain preventive behaviors among the public, evidence on social, cognitive, and psychological factors associated with the behaviors is necessary. Prior studies on infectious disease epidemics showed that knowledge and awareness [9–11], risk perception [7, 10, 12], and efficacy belief [6] help motivate people to adopt preventive behaviors. Similarly, recent studies on COVID-19 revealed that knowledge [13–16], perceived controllability [14, 17], optimistic beliefs [15, 17], emotion [15], and risk perception [16] might all account for precautionary actions of the public. To date, several KAP studies have examined the associations of knowledge with attitudes or practices beyond understanding the prevalence of each. The results of these previous studies revealed that a higher level of knowledge is positively related to the practice of preventive measures [18–22], and attitudes also associate positively with preventive behaviors [18, 20–22]. However, most of these studies examined the direct effects of knowledge on practicing preventive behaviors or attitudes without exploring the indirect effects of knowledge on practices mediated via attitudes to explicate the in-depth psychological mechanism behind how individuals perform behaviors based on their health knowledge. Specifically, how knowledge affects practices indirectly via attitudes in the context of COVID-19 is still less known.

When public health interventions and policies aim to protect health for all, attention should be given to vulnerable populations. KAP surveys are commonly used to identify knowledge gaps and behavioral patterns among sociodemographic subgroups to implement effective public health interventions [18]. The issue of health inequalities unfolding during disease outbreaks has been extensively investigated across pandemics [23, 24]. For example, the novel influenza A (H1N1) burden was substantially higher for people who were less educated [25], living in more deprived neighborhoods [25, 26], and experiencing more significant financial barriers [27]. In the case of the 2015 Middle East Respiratory Syndrome (MERS), Lee et al. have shown social determinants were directly (gender, education) or indirectly (age, education, income) related to practicing preventive behaviors [28]. Evidence of an unequal burden of COVID-19 is also emerging fast. People living in impoverished and racially and economically polarized areas showed considerably more significant morbidity and mortality rates of COVID-19 [29].

It is also worth noting that the COVID-19 burden may be coupled with existing non-communicable diseases among marginalized social groups, particularly those in minority ethnic groups, socioeconomic deprivation, and poverty, aggravating the populations’ overall vulnerability [23, 29, 30]. Behavioral factors related to COVID-19 are also unevenly distributed among people [31]. Studies have shown that males, less educated individuals, and the elderly showed lower COVID-19 knowledge and behaviors than their counterparts [19], and risk perception varied by the level of social support [6]. Another study on Chinese undergraduate students revealed that gender, major,affect students’ attitudes and practices [32]. Given these alarming inequalities in behavioral factors, there remains an urgent need to identify vulnerable populations during the COVID-19 pandemic to ensure health education and communication interventions tailored for their needs.

There is limited evidence concerning behavioral factors and related vulnerability during the COVID-19 pandemic in South Korea. The present study addresses whether the public performs precautionary behaviors recommended by the national guideline and behavioral interventions, along with which populations to prioritize in health behavior change interventions. We quantified and tested the relationships between knowledge, attitudes, and practices and examined how sociodemographic characteristics interplay with the behavioral components. Specifically, this study (1) investigates the level of knowledge, attitudes, and practices toward COVID-19, (2) explores how COVID-19 knowledge influences practices and whether the relationship is mediated by attitudinal factors (risk perceptions and efficacy beliefs), and (3) identifies which populations show low levels of knowledge toward COVID-19, making them highly likely to remain vulnerable during the pandemic. Implications for developing and implementing evidence-based health behavior interventions and policies during the COVID-19 pandemic are also discussed in this paper.

## Methods

### Study participants and survey

We adopted a cross-sectional survey design to evaluate the public’s knowledge, attitudes, and practices during the COVID-19 epidemic using an anonymous online questionnaire (see Additional file 1 for details). The survey was conducted via an online platform from a research company called Korea Research. The company recruited respondents by sending survey invitations containing general information about the survey, including its purpose and consent statement via e-mail or text messages, to registered nationwide survey panel members who met the inclusion criteria. The criteria required that each participant be: (1) aged 18 years or older, (2) a resident in South Korea, and (3) a Korean speaker. The company enrolled respondents using age, sex, and geographic region-based proportional and quota sampling process. Over 1000 subjects completed the surveys (55.2% response rate), and 970 were included in the analysis after excluding incomplete responses. The data collection took place over 3 days (June 26–29), 5 months after the Korea Centers for Disease Control and Prevention (KCDC) confirmed the first case at the early stage of the epidemic, and there have been 12,602 confirmed cases and 282 deaths as of June 26.

### Measurements

Respondents’ knowledge about COVID-19 was assessed using a six-item questionnaire developed by Zhong et al. [17] and adopted by other similar studies [33, 34]. The questionnaire included three questions about clinical characteristics of the disease (i.e., primary symptoms, availability and effectiveness of treatment, and severity). Two survey questions addressed transmission (i.e., infection through contact with animals and transmission through respiratory droplets), and one item covered prevention and control (i.e., wearing medical masks for prevention). All respondents could respond, “Yes,” “No,” or “Don’t know.” The knowledge scores were calculated by assigning one point to each correct question, and an aggregate score was calculated (range 0–6), with higher scores indicating more knowledge about COVID-19.

Precautionary behavior practices were measured using three items that covered the following two categories: (1) preventive measures (i.e., wearing facial masks and practicing hand hygiene) and (2) social distancing (i.e., avoiding crowded places). Respondents self-reported the frequency of the practices performed during the previous week at the time of the survey, using a 4-point Likert-type scale (1 = never, 2 = sometimes, 3 = often, and 4 = always).

To measure attitudes related to COVID-19, we examined the perceived risk of COVID-19 infection (two items) comprising perceived susceptibility, which signifies individuals’ beliefs about their possibility of infection, and perceived severity of the infection [35]. Respondents answered, “What do you think is the possibility of your COVID-19 infection?” and, “What do you think will be the severity if COVID-19 infects you?” Responses were rated on a 5-point Likert-type scale, with “1 = very low, 3 = neither low nor high, and 5 = very high” (Table 4). Concerning efficacy beliefs, respondents answered, “To what extent do you think the precautionary behavior is an effective way to reduce the risk of COVID-19 infection?” Each category of practice was tested in this study: ‘practicing personal hygiene such as wearing facial masks and hand hygiene’ or ‘social distancing such as avoiding crowded places.’ The responses were rated on a 4-point Likert-type scale ranging from 1 to 4 (1 = not at all and 4 = extremely) (Table 4).

Sociodemographic factors included gender, age, marital status and the presence of children younger than elementary school at home. We also assessed level of education and monthly household income in South Korean won [KRW]. We also collected information about the respondents’ residence (Table 1) .

**Table 1:** Descriptive statistics of survey respondents (*n* = 970)

Sociodemographic characteristics	Total (*n =* 970)	
	***n***	%
**Gender**		
Male	471	48.6
Female	499	51.4
**Age (year)**	*M = 47.44*	*SD = 14.78*
18–29	164	16.9
30–39	154	15.9
40–49	185	19.1
50–59	195	20.1
≥ 60	272	28
**Education level**		
High school	574	59.2
Bachelor’s degree	309	31.9
Graduate/professional degree	87	9
**Monthly household income**		
< 200	137	14.1
200–399	300	30.9
400–599	260	26.8
≥ 600	273	28.1
**Marital status**		
Married	604	62.3
Single/divorced/bereaved	366	37.7
**Presence of children**		
None	743	76.6
More than 1	227	23.4
**Residence**		
Urban	847	87.3
Rural	123	12.7

### Ethical considerations

Approval of the Seoul National University Institutional Review Board was obtained before conducting the study (IRB No. 2006/003–023). The respondents provided electronic informed consent that appeared on the first page of the survey by answering a “Yes or No” question before being allowed to complete the online self-reporting questionnaire. The company which conducted the online survey protected the confidentiality of anonymous respondents.

### Statistical analyses

We conducted statistical analyses using R version 4.0.2 (R Foundation for Statistical Computing, Vienna, Austria). All results of quantitative variables were reported either as mean (M), standard deviation (SD), or frequency (percentage %). Multivariate linear regression analysis was performed. Additionally, the indirect effects of knowledge on practices via attitudes were calculated using PROCESS model 6 macro with 5000 bootstrap samples for IBM SPSS Statistics 25 [36]. The bias-corrected 95% confidence intervals for each mediational path were obtained.

## Results

### Sociodemographic characteristics

One-thousand individuals responded to the survey, and after excluding individuals with missing data, 970 individuals were included in the final analyses (Table 1). The average age of participants was 47.44 years (SD =14.78). Approximately half were females (51.4%), and 59.2% had a high school diploma, followed by a bachelor’s degree (31.9%) and graduate or professional degree (9%). Additionally, 30.9% of the respondents’ monthly household income was within 200–400 (10,000 KRW), followed by over 600 (28.1%), 400–600 (26.8%), and under 200 (14.1%). Most were married (62.3%), had no children (76.6%), and resided in urban areas (87.3%) (Table 1).

### Knowledge, attitudes, and practices concerning COVID-19

Most respondents answered about four of six knowledge items correctly (*M* = 4.21, *SD* = 1.16). Respondents appeared to be knowledgeable about transmission through respiratory droplets of infected people (93.2% answered correctly, 2.5% incorrectly, and 4.3% reported that they did not know). The high prevalence of misunderstanding was discovered in a knowledge item, with participants believing that infection could occur through eating or having contact with wild animals (Table 2). Only 27.9% correctly answered the statement was false, 42.2% believed it was true, and 29.9% said they were not sure. About half of the respondents (48.8%) replied that wearing a general medical mask helps prevention was correct, but 39.7% answered incorrectly, and 11.5% did not know.

**Table 2:** Responses to knowledge items (%)

#	Knowledge items	Correct	Incorrect	Do not Know
1	The main clinical symptoms of COVID-19 are fever, fatigue, dry cough, and myalgia.	85.1	7.8	7.1
2	There currently is no effective cure for COVID-2019, but early symptomatic and supportive treatment can help most patients recover from infection.	76.5	9.7	13.8
3	Not all persons with COVID-2019 will develop severe cases. Only those who are elderly have chronic illnesses are more likely to be in severe cases.	89.3	6.7	4
4	Eating or contacting wild animals would result in infection by the COVID-19 virus.	27.9	42.2	29.9
5	The COVID-19 virus spreads via respiratory droplets of infected individuals.	93.2	2.5	4.3
6	Ordinary residents can wear general medical masks to prevent infection by the COVID-19 virus.	48.8	39.7	11.5

The knowledge score varied by gender and education levels (Table 2). Females (*β* = 0.14, *p* <  0.05) and individuals with higher levels of education (*β* = 0.06, *p* <  0.05) were more likely to have accurate information about COVID-19 (Table 3). Respondent’s age, income level, marital status, residence, and presence of children were not related to the knowledge level of COVID-19.

**Table 3:** Sociodemographic determinants of COVID-19 knowledge

	B	Std. Error	Beta	t	***p***-Value
Constant	3.58	0.38		9.52	< 0.001
Gender^a^	0.14	0.07	0.06	1.93	0.04
Age	0.05	0.03	0.07	1.61	0.11
Education level	0.06	0.03	0.06	1.9	0.04
Income level	0.008	0.04	0.008	0.05	0.96
Marital status	−0.02	0.11	−0.01	− 0.15	0.88
Residence^b^	0	0.11	0	0.04	0.96
Presence of children^c^	0.09	0.1	0.03	0.89	0.37
Adjusted R^2^	0.013				

^a^Male:1, Female:2

^b^City:1, Town:2

^c^Yes:1, None:0

Respondents perceived the risk of becoming infected with COVID-19 (perceived susceptibility) as lower than “neither high nor low” (score = 3) (*M =* 2.77, SD = 0.80); the average perceived severity score was higher than perceived susceptibility, which was close to “high” (score = 4) (*M =* 3.77, *SD =* 0.85). Both efficacy beliefs on preventive measures (*M =* 3.82, *SD =* 0.44) and social distancing (*M =* 3.66, *SD =* 0.59) were high. The most frequently performed practice was wearing facial masks (*M =* 3.82, *SD =* 0.49), followed by practicing hand hygiene (*M =* 3.51, *SD =* 0.66) and social distancing (*M =* 3.11, *SD =* 0.90) (Table 4).

**Table 4:** Responses of knowledge, attitudes, and practices

Variable	Range	***M***	***SD***
**Knowledge**			
Knowledge score	0–6	4.21	1.16
**Attitudes**			
*Perceived risk*			
Perceived susceptibility	1–5	2.77	0.80
Perceived severity	1–5	3.77	0.85
*Efficacy belief of precautionary behavior*			
Practicing personal hygiene	1–4	3.82	0.44
Avoiding crowded places	1–4	3.66	0.59
**Practices**			
Wearing facial masks	1–4	3.82	0.49
Practicing hand hygiene	1–4	3.51	0.66
Avoiding crowded places	1–4	3.11	0.9

### The influence of knowledge on attitudes

The role of knowledge in perceived susceptibility, severity, and efficacy belief was examined using regression analyses (Table 5). After controlling for sociodemographic factors, those who have less knowledge (*β* = − 0.12, *p* <  0.001) were more likely to have lower levels of perceived susceptibility of COVID-19. Respondents with higher knowledge displayed higher efficacy beliefs for personal hygiene practices (*β* = 0.19, *p* <  0.001), such as wearing masks and practicing hand hygiene, and had higher efficacy beliefs for avoiding crowded places (*β* = 0.16, *p* <  0.001) (Table 5).

**Table 5:** Determinants of attitudes toward COVID-19

	Perceived susceptibility of COVID-19			Perceived severity of COVID-19			Efficacy belief on personal hygiene			Efficacy belief on social distancing		
	B	Beta	*p*-value	B	Beta	*p*-value	B	Beta	*p*-value	B	Beta	*p-*value
Constant	2.99		< 0.001	3.41		< 0.001	3.27		< 0.001	3		< 0.001
Gender^a^	0.15	0.09	< 0.001	0.15	0.09	0.01	0.08	0.09	< 0.001	0.09	0.08	0.02
Age	0.02	0.04	0.3	0.09	0.15	< 0.001	0.03	0.11	0.01	0.05	0.12	< 0.001
Education level	−0.06	− 0.05	0.12	− 0.04	− 0.03	0.41	− 0.03	− 0.04	0.24	− 0.07	− 0.08	0.02
Income level	0.01	0.02	0.61	−0.05	−0.06	0.1	0.001	0.001	0.99	0	0	0.97
Marital status	0.04	0.02	0.58	−0.04	−0.02	0.63	−0.03	−0.03	0.49	−0.04	− 0.03	0.49
Residence^b^	−0.21	−0.09	0.01	−0.03	− 0.01	0.74	0.04	0.03	0.29	0.1	0.06	0.06
Presence of children^c^	0.19	0.1	< 0.001	0.12	0.06	0.1	0.03	0.03	0.35	0.09	0.06	0.07
Knowledge score	−0.08	−0.12	< 0.001	0.01	0.009	0.93	0.07	0.19	< 0.001	0.08	0.16	< 0.001
Adjusted R-squared	0.01			0.03			0.06			0.06		

^a^Male:1, Female:2

^b^City:1, Town:2

^c^Yes:1, None:0

### The influence of knowledge and attitudes on practices

Three different preventive behaviors varied by the knowledge and attitudes of the respondents (Table 6). First, those with higher efficacy beliefs (*β* = 0.31, *p* <  0.001) were more likely to wear a facial mask. Next, those with higher perceived susceptibility (*β* = 0.08, *p* <  0.05) and efficacy beliefs (*β* = 0.20, *p* <  0.001) were more likely to practice hand hygiene. Lastly, individuals who showed higher efficacy beliefs (*β* = 0.22, *p* <  0.001) tended to avoid crowded places to prevent COVID-19. Efficacy beliefs have shown the strongest and significant effects on behaviors; however, knowledge has not shown significant direct effects on the three practices (Table 6).

**Table 6:** Determinants of preventive behaviors toward COVID-19

Variables	Wearing facial masks			Practicing hand hygiene			Avoiding crowded places		
	B	Beta	***p***-Value	B	Beta	***p***-Value	B	Beta	***p***-Value
Constant	2.13		< 0.001	1.86		< 0.001	1.66		< 0.001
Gender^a^	0.1	0.1	< 0.001	0.15	0.12	< 0.001	0.02	0.01	0.78
Age	0.001	0.001	0.98	0.01	0.01	0.77	0.12	0.19	< 0.001
Education level	0.02	0.07	0.04	0.01	0.02	0.45	−0.03	− 0.04	0.26
Income level	−0.008	− 0.01	0.76	− 0.01	− 0.01	0.81	0.008	0.01	0.98
Marital status	0.04	0.04	0.32	0.06	0.04	0.33	−0.03	−0.02	0.69
Residence^b^	−0.06	−0.04	0.19	−0.08	− 0.04	0.22	0.07	0.03	0.37
Presence of children^c^	−0.03	−0.03	0.42	0.03	0.02	0.61	0.02	0.01	0.78
Knowledge score	0.007	0.01	0.73	0.01	0.02	0.52	−0.05	−0.06	0.05
Perceived susceptibility	0.03	0.04	0.2	0.06	0.08	0.03	0.02	0.01	0.68
Perceived severity	0.02	0.03	0.29	0.007	0.008	0.98	−0.04	−0.03	0.29
Efficacy belief	0.35	0.31	< 0.001	0.30	0.20	< 0.001	0.34	0.22	< 0.001
Adjusted R^2^	0.12			0.06			0.09		

^a^Male:1, Female:2

^b^City:1, Town:2

^c^Yes:1, None:0

### Relationship between knowledge, attitudes, and practices

The indirect effects of knowledge on preventive behaviors mediated by attitudes (efficacy) were significant (Table 7). Efficacy beliefs significantly mediated the relationship between knowledge and all three preventive behaviors—wearing a facial mask, practicing hand hygiene, and avoiding crowded places. However, perceived susceptibility negatively mediated the relationship between knowledge and practicing hand hygiene behavior.

**Table 7:** Indirect effects of knowledge on practices via attitudes

Dependent Variable	Mediator	Est.	95% BC^**a**^
Wearing facial masks	Perceived susceptibility	−0.0021	[− 0.0064, 0.0015]
	Perceived severity	0.0001	[−0.0015, 0.0014]
	Efficacy belief	0.0253	[0.0126, 0.0401]
Practicing hand hygiene	Perceived susceptibility	−0.001	[− 0.0018, 0.0002]
	Perceived severity	0.0002	[−0.0013, 0.0015]
	Efficacy belief	0.0218	[0.0101, 0.0362]
Avoiding crowded places	Perceived susceptibility	−0.0013	[− 0.0081, 0.0047]
	Perceived severity	0.0001	[−0.0025, 0.0026]
	Efficacy belief	0.0274	[0.0140, 0.0429]

Note. Unstandardized point estimates (Est.) are the indirect effect of the IV on the DV through the mediator

*BC*^a^ bias-corrected confidence interval; 95% bias-corrected confidence interval does not cross zero, and thus, mediation is assumed

## Discussion

Our findings demonstrated that the respondents have adequate knowledge about COVID-19, including the transmission of the virus through respiratory droplets of infected people and clinical symptoms of the disease. The perceived risk for infection susceptibility was relatively lower than the disease’s perceived severity regarding attitudes. The impact of efficacy beliefs on preventive measures was high in both personal hygiene and social distancing. Most respondents complied with the recommended practices such as wearing facial masks, practicing hand hygiene, and social distancing to prevent COVID-19 infections.

Several findings on the associations among KAP factors provided valuable insights into how public health initiatives can better protect the population’s health during public health emergencies, such as emerging infectious disease pandemics, by establishing strategic behavioral interventions. First, knowledge can play a crucial role in enhancing the practice of public preventive behavior, as our findings showed that knowledge was associated with attitudes and preventive behaviors. Others have previously reported similar associations when performing KAP surveys toward COVID-19 [18–20, 34]. This result implies that information disseminated through health interventions to prevent and control epidemics must be based on scientific evidence and delivered in understandable language to heighten public knowledge of the issues [37]. Although it is difficult to say how much knowledge is sufficient enough for achieving desirable changes in health outcomes, the impact of knowledge on health behaviors has been validated in many public health areas [9, 19, 38] based on the premise that the public can make ‘informed decisions’ about health behaviors by leveraging their knowledge about relevant health issues. While there are numerous definitions of informed decision-making [39–41], they commonly agree that informed decisions are based on sufficient knowledge of scientific evidence about the relevant aspects of the available alternatives [42].

In addition to providing sufficient and precise information, efforts to correct inaccurate and misguided information are needed. The “infodemic” phenomenon refers to an overabundance of information—potentially invalid or harmful information—spread on the internet or through other media. The infodemic is a tremendous and ongoing challenge during the COVID-19 pandemic [43–45]. Information production and consumption have increased significantly since the start of the pandemic, meaning the public is more easily exposed to misinformation [46–48]. During health crises, engaging the public with behavior change initiatives may be profoundly limited when disseminated health information conflicts with existing beliefs stemming from culture and system [49, 50], and rumors or misinformation are rampant across communication sources [51]. We recommend that public health practitioners and policymakers promote knowledge and understanding while addressing contextual factors that may hinder the public’s learning processes concerning health information. Notably, this study found a high prevalence of misunderstanding regarding the source of infection through eating or contact with wild animals, as only 27.9% of respondents correctly answered the information was false. Our study did not delve into the contexts behind this misinformation. Thus, we suggest that future research identify and monitor such misconceptions about COVID-19 dispersed across communication platforms to provide accurate and evidence-based information about the disease and prevention measures.

Second, attitudes, especially efficacy beliefs, had a significant and robust impact on practicing preventive behaviors, implying that promoting preventive behaviors toward COVID-19 would require promoting both knowledge and efficacy beliefs among the public. Consistent with evidence that efficacy beliefs serve as significant predictors of preventive behaviors [6, 48, 52, 53], this study also displayed that for the public to perform precautionary behaviors after acquiring information, they then need to believe that such practices would be effective. For example, people need to believe that washing hands would keep them from being infected, beyond merely informed so, to perform and sustain the behavior. While knowledge itself is at the root of learning, a discrepancy between information delivered and received is expected, given individual characteristics [54, 55]. Public health experts need to acknowledge that health communication is a dynamic process shaped mainly by individual cognitive and psychological factors. Our findings imply that a particular emphasis should be placed on bolstering efficacy; thus, COVID-19 behavior programs may integrate messaging strategies that stress the effectiveness of target behaviors (e.g., estimated reduced risks after the uptake of practicing hand hygiene) promoted by the programs. We also recommend that the efforts prioritize populations who displayed low efficacy beliefs, particularly those who are younger and have less knowledge of COVID-19.

Third, our study results showed that COVID-19 knowledge, attitudes, and practice differed by sociodemographic factors. Specifically, males and less educated individuals had less knowledge about COVID-19, rendering them particularly vulnerable to the epidemic. This result is similar to prior research investigating the association between sociodemographic factors and knowledge level during the COVID-19 pandemic in China [34, 48] and Hong Kong [53]. Many health communications studies have examined the phenomena of knowledge inequality. Such studies have emerged, particularly since the knowledge gap hypothesis postulated that people would acquire knowledge at different paces, widening the knowledge gap over time, depending on their socioeconomic status, cognitive capabilities, and prior knowledge [52, 55–58].

This study did not explore the temporal trend of inequalities; nevertheless, it identified the gaps in all factors within a causal link. Substantial differences among the respondents were evident in knowledge, attitudes, and behaviors. Thus, reducing gaps in health behaviors and outcomes may be achieved by decreasing knowledge inequalities and prioritizing them with scant health knowledge. Attention must be paid to those who showed particularly low levels of COVID-19 knowledge, as they are less likely to have proper attitudes and perform preventive behaviors. When all behavioral aspects are disproportionately distributed across different social groups, future policies and interventions should not be one-size-fits-all, as shown in this study. We suggest public health authorities should attempt a ‘person-centered’ approach rather than a ‘disease-centered’ approach to investigate vulnerable subpopulations and prioritize policies and communication efforts to accommodate the underserved’s needs.

Several limitations of this study should be acknowledged. First, we used the average score of knowledge in the analysis, so each knowledge item’s effect was not examined. Second, our study did not extensively explore other attitudinal factors associated with COVID-19 behaviors, such as perceived barriers or other communication factors that may have influenced the public’s knowledge, including seeking information, using the media, or processing information. Third, while efficacy beliefs can be conceptualized to include both response efficacy and self-efficacy, our study only adopted and examined the former, providing limited perspectives on the concept’s inherently composite nature.

## Conclusions

During health crises and emergencies, the public needs to practice precautionary behaviors at all times, as the novelty and unpredictability of epidemics may exceed a health system’s capability to a significant degree. This study provides evidence that knowledge is an essential predictor of attitudes and behaviors, contributing to advancing intervention strategies to promote and sustain the public’s precautionary behaviors in the context of the COVID-19 pandemic. Meanwhile, our study’s findings suggest that some people may be disadvantaged from performing health behaviors due to the unequal distribution of knowledge, attitudes, and behaviors, possibly in combination with a lack of access to health care and pre-existing health conditions. Finally, this study provides critical and timely insights into how the government and public health organizations establish and implement appropriate policies and interventions that do not overlook and deprioritize those in urgent need.

## Supplementary Information

**Additional file 1.** Survey questionnaire.

## Data Availability

The datasets used and analyzed in the current study are available from the corresponding author on reasonable request.

## Abbreviations

COVID-19: Coronavirus disease 2019
WHO: The World Health Organization
PHEIC: Public Health Emergency of International Concern
H1N1: Novel influenza A
MERS: Middle East Respiratory Syndrome
KCDC: Korea Centers for Disease Control and Prevention
KRW: Korean won
M: Mean
SD: Standard deviation
Est.: Estimates
BC: Bias-corrected confidence level
