# Supplementary material for: Knowledge, attitudes, and practices (KAP) toward COVID-19: a cross-sectional study in South Korea
Source: BMC Public Health. 2021 Feb 5;21:295. doi: 10.1186/s12889-021-10285-y (PMC7863060; doi:10.1186/s12889-021-10285-y)
Supplement: Supplementary file 1 — Additional file 1. Survey questionnaire. [file 12889_2021_10285_MOESM1_ESM.docx]

**Additional file 1. Survey questionnaire**

The information described below provides the questionnaire used in this study which was developed for this study.

**[Knowledge]**

| **#** | **Knowledge items** | **Yes** | **No** | **Do not know** |
| --- | --- | --- | --- | --- |
|  | **Are the following facts correct?** | | | |
| K1 | The main clinical symptoms of COVID-19 are fever, fatigue, dry cough, and myalgia. | 1 | 2 | 9 |
| K2 | There currently is no effective cure for COVID-2019, but early symptomatic and supportive treatment can help most patients recover from infection. | 1 | 2 | 9 |
| K3 | Not all persons with COVID-2019 will develop severe cases. Only those who are elderly have chronic illnesses are more likely to be in severe cases. | 1 | 2 | 9 |
| K4 | Eating or contacting wild animals would result in infection by the COVID-19 virus. | 1 | 2 | 9 |
| K5 | The COVID-19 virus spreads via respiratory droplets of infected individuals. | 1 | 2 | 9 |
| K6 | Ordinary residents can wear general medical masks to prevent infection by the COVID-19 virus. | 1 | 2 | 9 |

**[Attitudes]**

| # | **Perceived Risk of COVID-19 infection** | **Very low** |  | **Neither low nor high** |  | **Very high** |
| --- | --- | --- | --- | --- | --- | --- |
| A1 | What do you think is the possibility of your COVID-19 infection? | 1 | 2 | 3 | 4 | 5 |
| A2 | What do you think will be the severity if COVID-19 infects you? | 1 | 2 | 3 | 4 | 5 |

| # | **Efficacy beliefs** | **Not at all** |  |  | **Extremely** |
| --- | --- | --- | --- | --- | --- |
|  | **To what extent do you think the precautionary behavior is an effective way to reduce the risk of COVID-19 infection?** | | | | |
| A3 | Practicing personal hygiene such as wearing facial masks and hand hygiene’ | 1 | 2 | 3 | 4 |
| A4 | Social distancing such as avoiding crowded places. | 1 | 2 | 3 | 4 |

**[Practices]**

| # | **Practices of preventive behavior** | **Never** | **Sometime** | **Often** | **Always** |
| --- | --- | --- | --- | --- | --- |
|  | **In the last week, how often did you practice the followings?** | | | | |
| P1 | Wearing facial masks | 1 | 2 | 3 | 4 |
| P2 | Wash hands frequently and use hand sanitizer | 1 | 2 | 3 | 4 |
| P3 | Avoid visiting crowded places | 1 | 2 | 3 | 4 |

**[Backgrounds]**

B1. What gender do you identify as?

1. Male
2. Female

B2. What is your age?

( ) years

B3. What is the highest degree or level of school you have completed?

1. Middle school
2. High school
3. Some college education
4. Bachelor’s degree
5. Some graduate education
6. Master’s degree, professional degree or higher

B4. What is your marital status?

1. Married
2. Single
3. Divorced
4. Bereaved

B5. Which of these describes your monthly household income last year?

1. Less than 200 million KRW
2. 200 million to 399 million KRW
3. 400 million to 599 million KRW
4. 600 million KRW or above

B6. Do you have any children younger than elementary school at home?

1. Yes
2. No

B6. Which of these describes your current residence?

1. Urban
2. Rural
